# Image-guided puncture for differentiating malignant from benign peritoneal lesions: a systematic review and meta-analysis

**DOI:** 10.1007/s00330-025-12026-w

**Published:** 2025-11-20

**Authors:** Yi-Lin Hou, Jia-Yue Sun, Xue-Mei Wang, Zhi-Guang Chen, Xi-Yu Zhang, Cheng-Fei Sun, Di Wu, Yun-Fei Zhang

**Affiliations:** 1 Department of Ultrasound, The First Hospital of China Medical University, Shenyang City, China; 2 Class 3, Grade 2023, Science High School Department, Northeast Yucai School, Shenyang City, China; 3 Department of Ultrasound, General Hospital of Fushun Mining Bureau of Liaoning Health Industry Group, Shenyang City, China; 4 Department of Ultrasound, Liaoning Cancer Hospital & Institute, Shenyang City, China

**Keywords:** Peritoneum, Image-guided, Differential diagnosis, Biopsy, Fine-needle

## Abstract

**Objectives:**

To evaluate the diagnostic performance of image-guided puncture in differentiating malignant from benign peritoneal lesions.

**Materials and methods:**

An independent literature search was conducted across multiple English medical databases, including PubMed, Embase, Web of Science, Cochrane Library and Ovid. The diagnostic accuracy of image-guided puncture was compared against postoperative pathology and diagnostic laparoscopy, which served as reference standards. The diagnostic performance of imaging-guided puncture was evaluated by calculating pooled sensitivity, specificity, diagnostic odds ratio (DOR), and the area under the curve (AUC). Subgroup analyses were conducted based on imaging modality, type of puncture, and the risk assessment derived from the Quality Assessment of Diagnostic Accuracy Studies-2 (QUADAS-2).

**Results:**

Fifteen eligible studies, comprising a total sample of 1208 patients with 1165 peritoneal lesions, were included in the analysis. The pooled sensitivity of image-guided puncture for differentiating malignant from benign peritoneal lesions was 93% (95% confidence intervals (CI): 91–95%), with a specificity of 95% (95% CI: 92–97%), a DOR of 149.02 (95% CI: 78.47–282.99), and an AUC of 0.97.

**Conclusion:**

Overall, this meta-analysis demonstrates that image-guided puncture is a minimally invasive and safe technique with high diagnostic accuracy, regardless of the imaging modality, puncture method, or lesion type (mass or diffuse infiltration). It offers a reliable alternative to invasive biopsy for diagnosing peritoneal lesions. Subgroup analysis revealed no significant differences in diagnostic efficacy across the evaluated parameters.

**Key Points:**

***Question*** *The diagnosis of peritoneal lesions remains challenging due to the inherent complexity and invasiveness of traditional diagnostic laparoscopy, as well as overlapping imaging features of routine imaging examinations.*

***Findings*** *Image-guided puncture is a minimally invasive and safe technique with high diagnostic accuracy, irrespective of the imaging modality used, the puncture method, or the type of peritoneal lesion.*

***Clinical relevance*** *Image-guided puncture is recognized as a safe, minimally invasive, and highly sensitive diagnostic tool, which can reduce patient discomfort compared to diagnostic laparoscopy. In most clinical scenarios, it represents a reliable and effective alternative to this invasive procedure.*

**Graphical Abstract:**

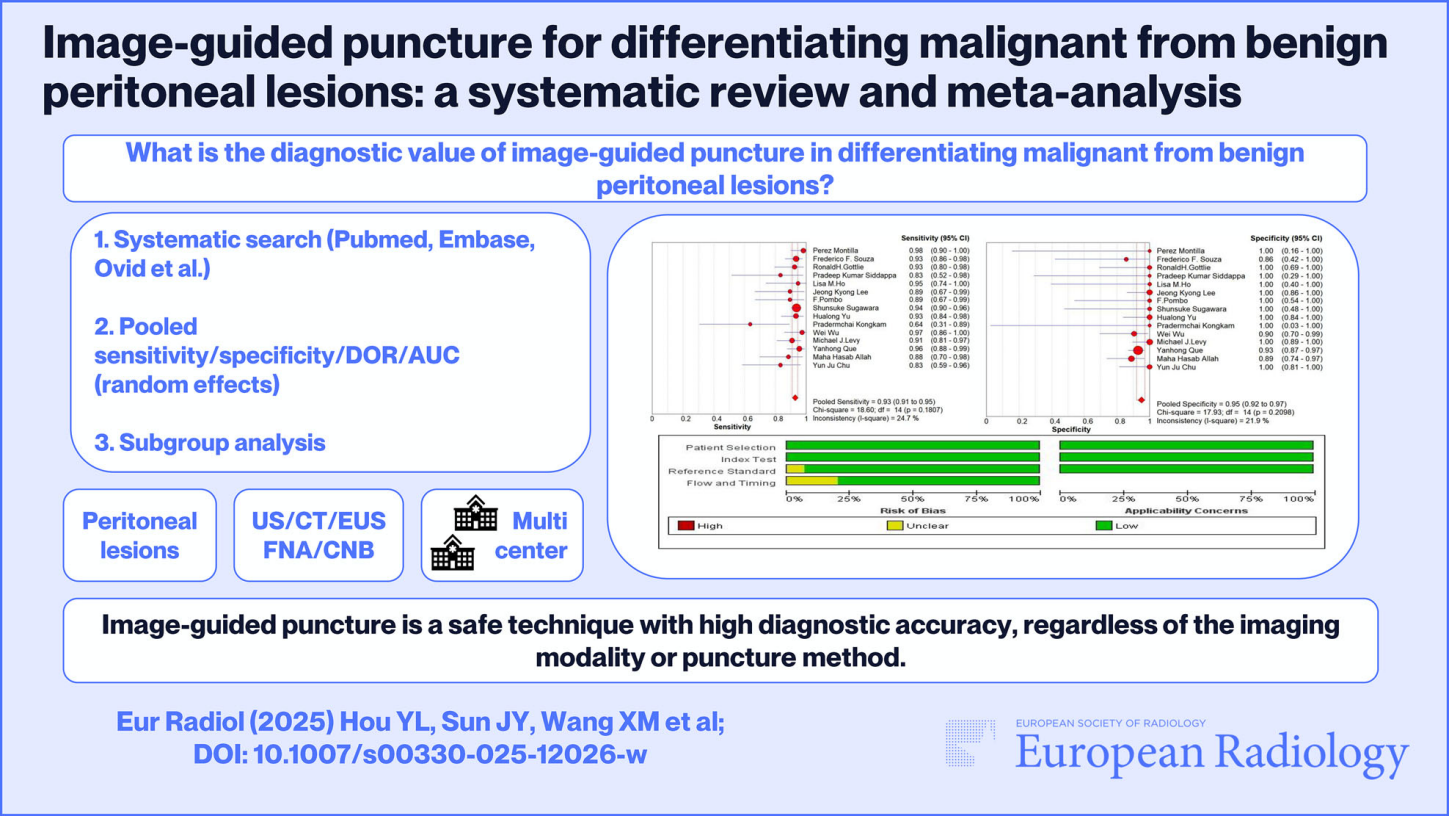

## Introduction

The peritoneum is the largest serosal membrane in the human body. Its parietal and visceral layers fuse to form structures such as the omentum and mesentery [1]. Peritoneal lesions encompass both benign conditions (e.g., tuberculous peritonitis) and malignant diseases (e.g., metastatic cancer), which are characterized by features such as peritoneal thickening, abdominal effusion and nodules [2, 3]. Conventional imaging modalities, including ultrasound (US), computed tomography (CT), and Magnetic resonance imaging (MRI), are commonly used for evaluation; however, benign and malignant peritoneal lesions often exhibit overlapping imaging features, making it challenging to distinguish between them based solely on imaging findings [4–6]. Traditional omental biopsy, performed via laparotomy or diagnostic laparoscopy, requires general anesthesia and carries risks such as infection and tumor dissemination during surgical investigation, which can result in significant patient morbidity [7–9].

Image-guided puncture can be performed using US, CT, or endoscopic ultrasound (EUS). Compared to traditional methods such as diagnostic laparoscopy or laparotomy, image-guided puncture is recognized as a safe, minimally invasive, and highly sensitive diagnostic tool, as increasingly evidenced in clinical practice [10–14]. Numerous studies have been published in recent years on the differentiation between benign and malignant peritoneal lesions using image-guided puncture; however, the reported results vary significantly, with diagnostic sensitivity ranging from 64% to 98% and specificity ranging from 86% to 100% [4, 9–12, 15–24]. Therefore, the purpose of this study is to conduct a meta-analysis to evaluate the diagnostic performance of image-guided puncture in differentiating benign from malignant peritoneal lesions.

## Materials and methods

### Literature search

This study was reported in accordance with the PRISMA guidelines [25]. Two researchers independently conducted a comprehensive literature search of PubMed, Embase, Web of Science, Cochrane Library, and Ovid English medical databases. Keywords such as ‘peritoneal,’ ‘omentum,’ ‘fine needle aspiration (FNA),’ and ‘biopsy’ were used to identify all relevant studies on the diagnostic value of image-guided puncture for malignant and benign peritoneal lesions. The detailed search strategies are provided in Table 1. Duplicate articles were manually removed. Additionally, unpublished studies were identified, but none met the eligibility criteria for inclusion. The literature search was updated until October 10, 2024.

**Table 1:** Search strategy of each database

Database	Strategy
PubMed	((((((“Peritoneum”[Mesh]) OR (peritoneum[Title/Abstract])) OR (peritoneal[Title/Abstract])) OR (omentum[Title/Abstract])) OR (omental[Title/Abstract])) OR (mesentery[Title/Abstract])) AND ((((“Biopsy”[Mesh]) OR (biopsy[Title/Abstract])) OR (fine needle aspiration[Title/Abstract])) OR (puncture[Title/Abstract]))
Embase and Medline	#1 peritoneum OR peritoneal OR omentum OR omental OR mesentery #2 (biopsy OR fine) AND needle AND aspiration OR puncture #3 #1 AND #2
Cochrane Library	#1 MeSH descriptor: [peritoneum] explode all trees #2 (peritoneum):ti,ab,kw OR (omentum):ti,ab,kw OR (omental):ti,ab,kw OR (peritoneal):ti,ab,kw OR (mesentery):ti,ab,kw (Word variations have been searched) #3 #1 OR #2 #4 MeSH descriptor: [biopsy] explode all trees #5 (biopsy):ti,ab,kw OR (fine needle aspiration):ti,ab,kw OR (puncture):ti,ab,kw (Word variations have been searched) #6 #4 OR #5 #7 #3 AND #6
Web of Science	((ALL = (peritoneum OR peritoneal OR omental OR omentum OR mesentery)) AND ALL = (biopsy OR fine needle aspiration OR punture))
Ovid	#1 (peritoneum OR peritoneal OR omentum OR omental OR mesentery).titl. #2 (biopsy OR fine needle aspiration OR puncture).titl. #3 #1 AND #2

### Inclusion and exclusion criteria

All articles were independently assessed by two researchers. The inclusion criteria were as follows: (1) the study was approved by an ethical committee or institutional review board; (2) the study evaluated the diagnostic value of image-guided puncture for differentiating benign and malignant peritoneal lesions; (3) postoperative pathological and/or diagnostic laparoscopy were used as the reference standard; and (4) complete study data were available to calculate true positive (TP), false positive (FP), false negative (FN), and true negative (TN) values. The exclusion criteria were as follows: (1) case reports, animal experiments, conference reports, articles published before 1990, and non-English publications were excluded; (2) for studies with insufficient data, the corresponding author was contacted via email to obtain missing data, and the study was excluded if no response was received within 15 days; and (3) if two or more studies were conducted by the same department, those with smaller patient sample sizes were excluded. All disagreements between the researchers were resolved through consensus.

### Data extraction

Two researchers independently extracted all relevant data. In cases of disagreement during the data extraction process, the study in question was reviewed and resolved through discussion, resulting in the inclusion of 15 eligible studies. The extracted data included the first author, study country, year of publication, patient age, sex distribution, number of patients, number of study cases, reference standard, final pathology type, imaging modality, puncture type, and TP, FP, FN, and TN. All disagreements were resolved through consultation.

### Quality assessment

The quality of the included studies was assessed using the QUADAS-2 tool. The QUADAS-2 tool is a widely used framework for evaluating the risk of bias and applicability of primary diagnostic accuracy studies. The tool comprises four domains: (1) patient selection, (2) index test, (3) reference standard, and (4) flow and timing. Each domain is assessed for risk of bias, with the first three domains also evaluated for applicability concerns. The basic content and structure of the QUADAS-2 tool are summarized in Supplementary Materials 1. For each domain, the risk of bias is judged as “yes,” “no,” or “unclear,” while applicability concerns are categorized as “low,” “high,” or “unclear.” The QUADAS-2 tool provides a structured and transparent framework, ensuring consistency and rigor in the assessment process [26]. Two researchers independently evaluated the quality of all included studies, and any discrepancies were resolved through consultation.

### Data analysis

**Fig. 1:**
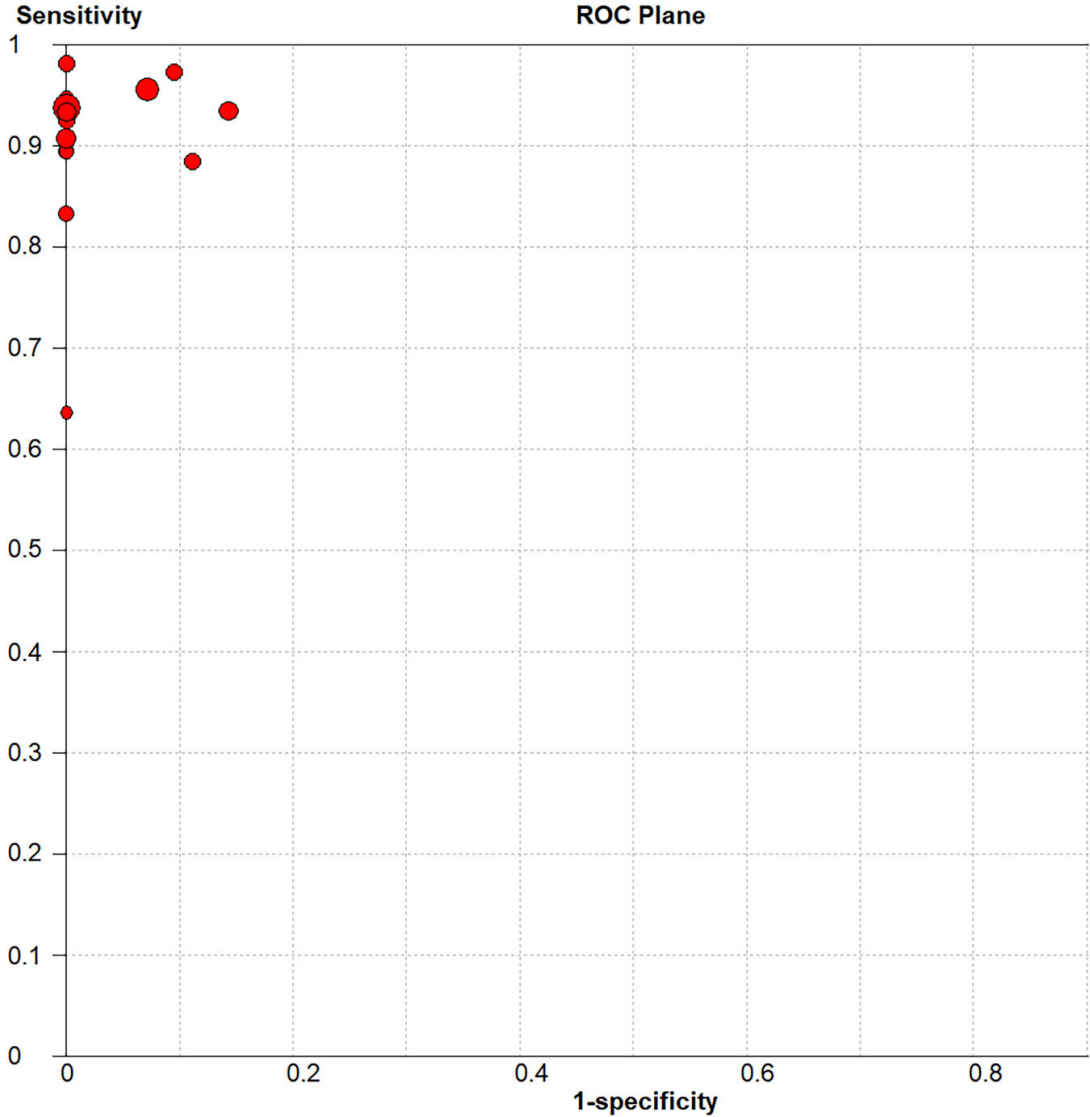
ROC plane of image-guided puncture for differentiating between malignant and benign peritoneum lesions

**Fig. 2:**
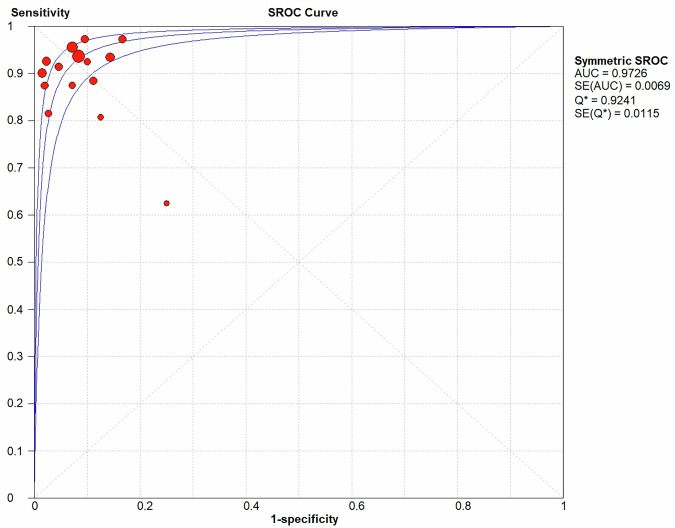
Summary receiver operating characteristic (SROC) curve on image-guided puncture for differentiating between malignant and benign peritoneum lesions. The middle curve is the SROC curve. The upper and lower curves show the 95% confidence intervals

Data analysis was performed using Meta-Disc (version 1.4), Review Manager (version 5.4), SPSS Statistics (version 17.0), and Stata (version 17). The Spearman correlation coefficient was used to assess threshold effects. Spearman correlation analysis was performed to assess the association between sensitivity and 1-specificity, which helps evaluate the presence of a threshold effect in the included studies. A strong positive correlation (ρ > 0.6) suggests the presence of a threshold effect, while a weak or absent correlation (ρ ≤ 0.6) indicates its absence. Heterogeneity was evaluated using the Cochran Q and i2 tests. In cases where zero values were observed for FP, FN, TP, or TN, 0.5 was added to these values to allow for the calculation of sensitivity and specificity. This adjustment ensures the stability of the summary receiver operating characteristic (SROC) curve analysis and avoids undefined values in logarithmic transformations. As a result, the points in Fig. 1 (unadjusted values) and Fig. 2 (adjusted values) may differ slightly. A random-effects model was used for all meta-analyses to account for potential heterogeneity among studies, as it provides a more conservative and realistic estimate of the effect size by considering both within-study and between-study variability. The fixed-effect model was not considered appropriate due to its strong assumption of a common effect size across studies, which is rarely justified in practice. Meta-Disc software was used to calculate pooled sensitivity, specificity, DOR, AUC, and the Q index, with 95% CI. Subgroup analysis was conducted to explore the diagnostic performance of image-guided puncture for differentiating benign and malignant peritoneal lesions. Publication bias was assessed using Deek’s funnel plot and Egger’s test in STATA, with a *p*-value < 0.05 indicating potential publication bias. The quality of the included studies was evaluated using the QUADAS-2 tool in Review Manager software. Interobserver agreement during article screening and QUADAS-2 application was analyzed using Cohen’s κ statistic in SPSS software.

## Results

### Literature search and characteristics of included studies

**Fig. 3:**
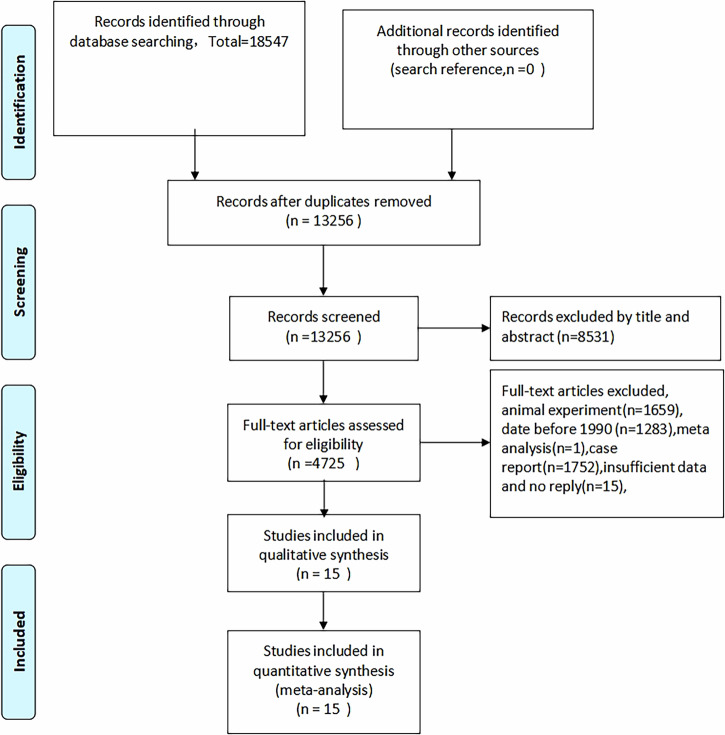
Flow diagram of study selection. *n* = number of studies

Following a comprehensive literature search, 18,547 articles were imported into EndNote. 15 studies were ultimately included after removing duplicates, categorizing, and screening titles and abstracts. The screening process is illustrated in Fig. 3. The included studies, published between 1996 to 2024, comprised 1165 cases of imaging-guided puncture for diagnosing benign and malignant peritoneal lesions. Table 2 details the specific characteristics of the included studies.

**Table 2:** Main characteristics of included studies

	Author	Country	Age (avg.)	Male/female	Diagnostic sample	Study population	Reference standard	Type of lesions (number of lesions)	Imaging modality	Puncture methods	TP	FP	FN	TN
1	M. E. Pérez Montilla et al	Spain	64	6/51	57	57	Postoperative pathology or clinical follow-up	Lymphoma (3), ovarian carcinoma (38), colon adenocarcinoma (3), endometrioid adenocarcinoma (3), pancreatic adenocarcinoma (1), breast carcinoma (1), gastrointestinal stromal tumor (1), gastric adenocarcinoma (2), appendicular carcinoma (1), nephroblastoma (1), fibrous tissue (1), acute inflammatory tissue (1)	CT combined with US	CNB	54	0	1	2
2	Frederico F. Souza et al	America	62	50/61	99	111	Postoperative pathology	Colorectal carcinoma (17), ovarian carcinoma (15), lymphoma (10), pancreatic carcinoma (6), liposarcoma (4), gastrointestinal stromal tumor (4), endometrial carcinoma (3), breast carcinoma (3), melanoma (3), leiomyosarcoma (3), vulvar cancer (2), others (41)	CT combined with US	FNA combined with CNB	86	1	6	6
3	Ronald H. Gottlie et al	America	52	13/39	50	52	Postoperative pathology or bone marrow aspiration	Lymphoma (8), ovarian adenocarcinoma (7), colonic adenocarcinoma (4), endometrial adenocarcinoma (4), others (27)	US	FNA combined with CNB	37	0	3	10
4	Pradeep Kumar Siddappa et al	India	64	4/11	15	54	Postoperative pathology	Ovarian carcinoma (4), gallbladder carcinoma (3), cholangiocarcinoma (2), others (6)	EUS	FNA	10	0	2	3
5	Lisa M. Ho et al	America	57	10/13	23	23	Postoperative pathology or specific pathologic diagnosis	Lymphoma (3), gastrointestinal stromal tumor (2), fibrosis (2), others (16)	US	FNA combined with CNB	18	0	1	4
6	Jeong Kyong Lee et al	Korea	52	21/24	44	45	Postoperative pathology or follow-up evaluation	Malignancy (19), tuberculosis (16), inflammation (9)	US	CNB	17	0	2	25
7	F. Pombo et al	Spain	56	7/18	25	25	Postoperative pathology or laparoscopic biopsy or other clinical methods	Metastatic adenocarcinoma (10), tuberculosis (5), pseudomyxoma peritonei (3), hepatocellular carcinoma (1), lymphoma (1), mesothelioma (1), actinomycosis (1), metastatic papillary serous carcinoma of the ovary (1), metastases from a hepatocellular carcinoma (1)	CT	CNB	17	0	2	6
8	Shunsuke Sugawara et al	Japan	64	67/230	311	297	Postoperative pathology or clinical follow-up	Peritoneal/ovarian/fallopian tube cancer (158), endometrial cancer (11), malignant mesothelioma (33), cancer of unknown origin (27), appendiceal cancer (9), malignant lymphoma (7), gist(5), colon cancer (6), cervical cancer (6), pseudomyxoma peritonei (5), endometrial stromal sarcoma (4), pancreatic cancer (4), lung cancer (2), malignant melanoma (3), esophageal cancer (2), gallbladder cancer (2), uterine carcinosarcoma (2), other malignant disease (14), inflammatory myofibroblastic tumor (2), other benign diagnosis (9)	CT combined with US	CNB	287	0	19	5
9	Hualong Yu et al	China	NA	26/58	81	84	Postoperative pathology	Metastases from ovarian cancer (44), metastases from gastrointestinal cancer (5), metastases from pancreatic cancer (1), unidentified primary lesions (5), malignant mesothelioma (4), lymphoma (1), pseudomyxoma peritonei (1), tuberculous peritonitis (11), inflammatory lesions (6)	CT	CNB	56	0	4	21
10	Pradermchai Kongkam et al	Thailand	63	6/6	12	12	Postoperative pathology	Pancreatic cancer (4), gallbladder cancer (1), gynecologic malignancy (3), cholangiocarcinoma (1), hepatocellular carcinoma (1), benign disease (1), carcinoma unknown primary (1)	EUS	FNA	7	0	4	1
11	Wei Wu et al	Chian	59	30/28	58	58	Diagnostic Laparoscopic Exploration or Final Clinical Diagnosis	Pancreatic cancer (5), gallbladder cancer (9), bile duct cancer (9), liver cancer (10), ovarian cancer (4), spontaneous bacterial peritonitis (7), tuberculous peritonitis (3), sclerosing peritonitis (2), fungal peritonitis (9)	EUS	FNA	36	2	1	19
12	Michael J. Levy et al	America	65	59/39	98	98	Postoperative pathology	Pancreatic carcinoma (50), cholangiocarcinoma (12), gastric carcinoma (2), pancreatic neuroendocrine tumor (3), pancreatic lymphoma (2), gallbladder carcinoma (2), duodenal carcinoma (1), cancer restaging (16), cancer restaging (10)	EUS	FNA	59	0	6	33
13	Yanhong Que et al	China	45	91/103	194	194	Postoperative pathology or clinical follow-up	Tuberculosis (121), carcinomatosis (68), chronic inflammation (3) others (2)	US	CNB	65	9	3	117
14	Maha Hasab Allah et al	Eygpt	41	24/38	62	62	laparoscopic biopsy	TB peritonitis (31), other inflammatory conditions (5), peritoneal carcinomatosis (19), Disseminated lymphoma (1), Primary malignant peritoneal pathology (6)	US	FNA combined with CNB	23	4	3	32
15	Yun Ju Chu et al	Korea	52	16/20	36	36	Postoperative pathology	Peritoneal carcinomatosis (18), tuberculous peritonitis (18)	US	CNB	15	0	3	18

### Quality assessment

**Fig. 4:**
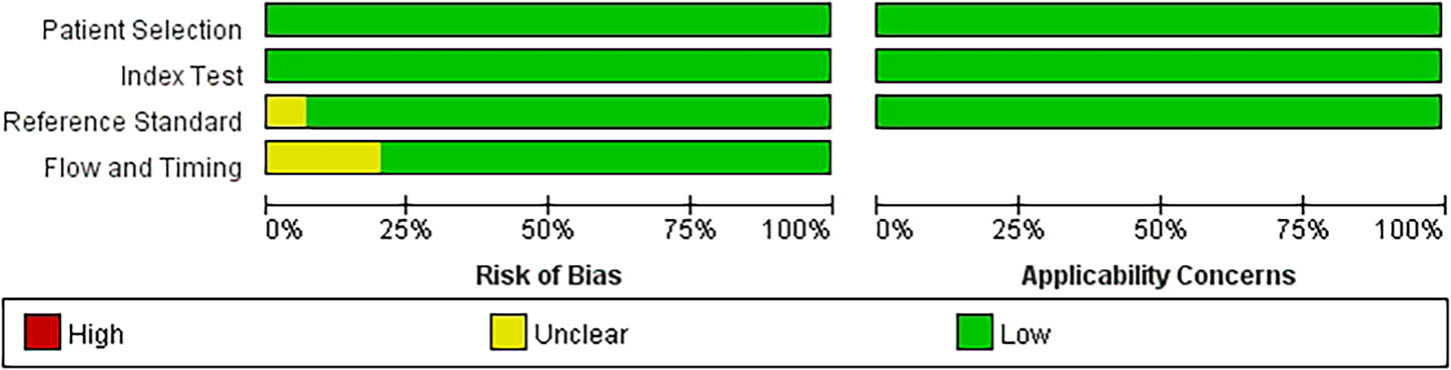
Quality assessment of the included studies using the QUADAS-2

Figure 4 displays the quality evaluation results of the included studies. Most indications across the studies demonstrated compliance, indicating a low overall risk of bias. However, it was unclear whether the pathologist was blinded to the image-guided puncture results in all the studies. Five studies did not include all cases: four due to a lack of specific pathological diagnosis [11, 16, 19, 21], and one due to the inclusion of subjects with associated ascites [22]. Another study reported a final pathologic diagnosis of probable malignancy [22]. Interobserver agreement was good (κ = 0.79; 95% CI: 0.62–0.95).

### Diagnostic accuracy for differential diagnosis between malignant and benign peritoneum lesions

**Fig. 5:**
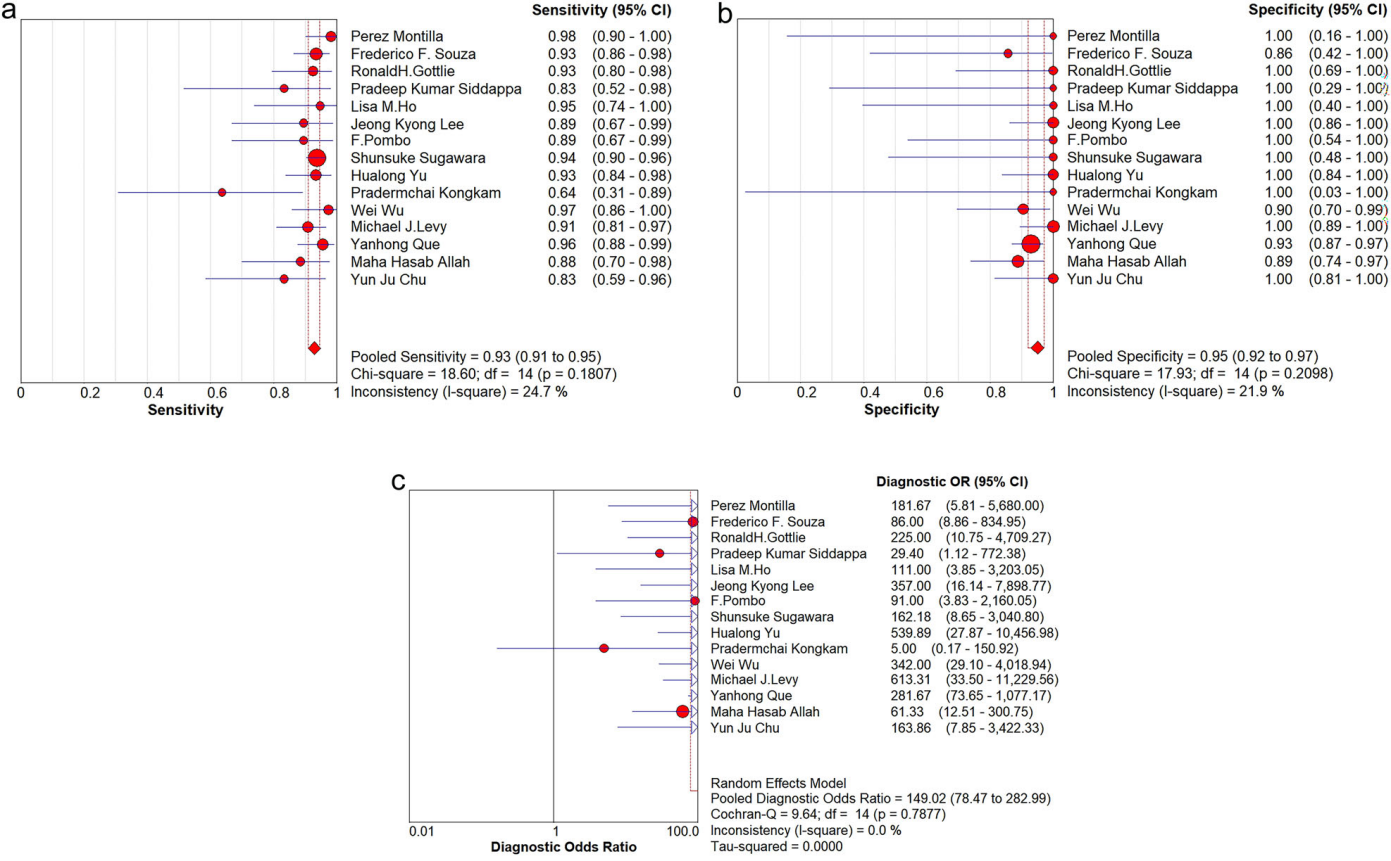
The pooled sensitivity (**a**), specificity (**b**) and DOR (**c**) of image-guided puncture for differentiating between malignant and benign peritoneum lesions

No heterogeneity was identified in the diagnostic threshold analysis, and no ‘shoulder-arm’ pattern was observed in the ROC plane (Fig. 1), with a Spearman correlation coefficient of 0.078 (*p* = 0.783). The diagnostic accuracy of image-guided puncture for differentiating malignant from benign peritoneal lesions was calculated, yielding a pooled sensitivity of 93% (95% CI: 91–95%), specificity of 95% (95% CI: 92–97%), DOR of 149.02 (95% CI: 78.47–282.99) (Fig. 5), and AUC of 0.973 (Q = 0.924) (Fig. 2).

### Subgroup analysis

Subgroup analyses were performed based on imaging modality, puncture technique, and QUADAS-2 risk assessment. The analyses revealed no statistically significant differences in diagnostic performance across subgroups (all *p*-values > 0.05, Table 3), with all absolute differences in sensitivity and specificity measures being < 5%. For imaging modalities, the pooled sensitivities ranged from 90% (EUS; 95% CI: 0.83–0.94) to 94% (CT combined with US; 95% CI: 0.92–0.96), while specificities ranged from 93% (CT combined with US; 95% CI: 0.66–1.00) to 100% (CT; 95% CI: 0.87–1.00). Among puncture techniques, core needle biopsy (CNB) demonstrated the highest sensitivity (94%; 95% CI: 0.91–0.96) and specificity (96%; 95% CI: 0.92–0.98), though these differences did not reach statistical significance. When stratified by QUADAS-2 assessment, both low-risk and unclear-risk study groups showed comparable diagnostic performance (sensitivity: 93% vs 92%; specificity: 94% vs 98%). Further details are provided in Table 3.

**Table 3:** Results of the subgroup analysis for differential diagnosis between malignant and benign peritoneum lesions

Subgroup	Number	Pooled sensitivity (95% CI)	Pooled specificity (95% CI)	Pooled DOR (95% CI)	AUC	*p*-value
Imaging modality						
US	6	0.92 (0.87–0.96)	0.94 (0.90–0.97)	168.70 (72.95–390.12)	0.974	0.266
CT	2	0.92 (0.84–0.97)	1.00 (0.87–1.00)	256.78 (31.37–2101.66)	NA	NA
EUS	4	0.90 (0.83–0.94)	0.97 (0.88–1.00)	115.17 (26.82–494.56)	0.959	0.200
CT combined with US	3	0.94 (0.92–0.96)	0.93 (0.66–1.00)	120.36 (24.83–583.47)	0.965	0.667
Puncture methods						
FNA	4	0.90 (0.83–0.94)	0.97 (0.88–1.00)	90.89 (11.14–741.80)	0.960	0.114
CNB	7	0.94 (0.91–0.96)	0.96 (0.92–0.98)	243.18 (96.96–609.93)	0.981	0.988
FNA combined with CNB	4	0.93 (0.88–0.96)	0.91 (0.81–0.97)	85.22 (27.58–263.33)	0.958	0.900
QUADAS-2 assessment						
Low risk	10	0.93 (0.91–0.95)	0.94 (0.90–0.97)	145.95 (67.04–312.72)	0.972	0.789
Unclear risk	5	0.92 (0.88–0.95)	0.98 (0.92–1.00)	176.89 (50.26–622.60)	0.967	0.624

*US* ultrsound, *EUS* endoscopic ultrasound, *CNB* core needle biopsy, *FNA* fine needle aspiration, *NA* not available

### Heterogeneity results

The Cochran Q and the I^2^ tests revealed no significant heterogeneity (*p* = 0.788 and I^2^ = 0.0%).

### Evaluation of publication bias

**Fig. 6:**
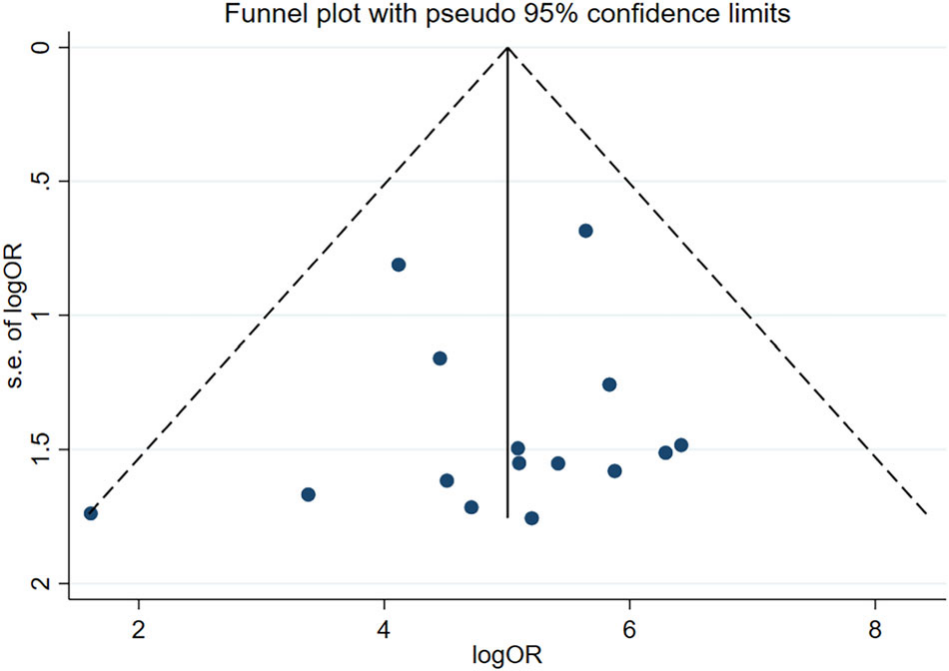
Deek’s funnel plot for evaluating potential publication bias

Publication bias was assessed using Deek’s funnel plot and Egger’s test. The distribution of Deek’s funnel plot was nearly symmetric, indicating no significant publication bias in this meta-analysis (*p* = 0.624) (Fig. 6).

## Discussion

Our meta-analysis of 15 studies (*n* = 1165 tissue samples) reveals image-guided peritoneal biopsy offers high diagnostic accuracy (sensitivity 93%, specificity 95%, AUC 0.973). The DOR of 149.02 (78.47–282.99) suggests strong discriminative ability, yet the wide CI reflects substantial variability across studies. A subgroup analysis was performed to evaluate the influence of various parameters on the diagnostic performance of image-guided puncture for differentiating benign and malignant peritoneal lesions (Table 3). Subgroup analysis based on imaging modality, puncture method, and QUADAS-2 risk assessment revealed no statistically significant differences in diagnostic performance. For imaging modalities, sensitivity ranged from 90% to 94%, and specificity from 93% to 100%. For puncture methods, sensitivity ranged from 90% to 94%, and specificity from 91% to 97%. For QUADAS-2 risk stratification, diagnostic performance was comparable between low-risk and unclear-risk groups, with sensitivities of 93% vs 92%, and specificities of 94% vs 98%, respectively.

First, the included studies were categorized into four groups based on the imaging modality used: US, CT, EUS and CT combined with US. The sensitivities for these groups were 92%, 92%, 90%, and 94%, respectively, while the specificities were 94%, 100%, 97%, and 93%, respectively. The results showed no significant differences in diagnostic performance among the four groups, suggesting that any of these imaging modalities can be effectively utilized. However, some differences persist among these imaging modalities in terms of their diagnostic capabilities and limitations. Our analysis included 1165 puncture cases, including 409 (35.1%) performed with US guidance [4, 16, 18–20, 23]. In the subgroup of peritoneal masses (*n* = 106), US-guided biopsies (*n* = 73, 68.9%) achieved excellent diagnostic performance, with a pooled sensitivity of 93.2% (95% CI: 86.4–97.2) and perfect specificity of 100% (95% CI: 94.2–100) [18, 19]. Similar high accuracy was observed for diffuse peritoneal thickening cases (*n* = 113; US-guided *n* = 80, 70.8%), though with slightly lower sensitivity (86.5%, 95% CI: 77.6–92.8) while maintaining perfect specificity (100%, 95% CI: 93.5–100) [4, 16]. Overall, US-guided puncture exhibited high diagnostic efficacy for both solitary peritoneal masses and diffuse thickening. The superior performance in mass lesions compared to diffuse lesions may be attributed to the fact that infiltrative lesions are typically less fixed and more challenging to target than discrete masses. Additionally, in early-stage carcinomatosis, inflammatory changes often predominate before overt mass formation; in such cases, the inflammatory component of peritoneal thickening may contribute to sampling errors during biopsy [4]. Moreover, US-guided peritoneal aspiration biopsy significantly improves diagnostic accuracy for differentiating benign from malignant ascites compared to conventional US signs alone, with sensitivity ranging from 88.5% to 95.6% and specificity from 88.9% to 92.9%. When combined with conventional US signs, specificity increases to 96.8% [20, 23].

However, US-guided puncture is more suitable for superficial peritoneal lesions, as intestinal gas interference can make it challenging to distinguish peritoneal nodules from intestinal structures. To address these limitations, a combined US and CT approach can be employed. In our study, this dual-modality strategy demonstrated the highest sensitivity of 94%. CT is particularly effective in detecting deep and subtle abnormalities, such as increased mesenteric density near the small intestine and enlarged lymph nodes [11]. For deep-seated or bowel-adjacent masses, CT localization can enhance US visualization by reducing intestinal gas interference. Similarly, in cases of mild infiltrative peritoneal thickening, contrast-enhanced CT helps identify the most severely affected areas, thereby improving the accuracy of subsequent US-guided biopsy [4, 16].

EUS-guided peritoneal aspiration biopsy has primarily been reported using FNA cytology. This technique is particularly relevant for diagnosing unexplained ascites, a persistent clinical challenge. Levy et al demonstrated EUS’s superiority in detecting small peritoneal implants and trace ascites that might evade detection by other imaging modalities. Further characterization of ascites can be achieved through FNA of adjacent peritoneal nodules [15, 17].

Second, the included studies were divided into three groups based on the puncture method: FNA, CNB, and FNA combined with CNB. The results demonstrated that CNB achieved the highest sensitivity and diagnostic efficacy, with a sensitivity of 94%, specificity of 96%, DOR of 243.18, and AUC of 0.981. Among the 889 cases where CNB was performed, 119 (13.4%) involved infiltrative lesions. Notably, multiple studies [4, 16, 21] have established imaging-guided CNB (via US or CT) as a highly specific diagnostic tool for peritoneal lesions, particularly during initial evaluation of mild diffuse peritoneal thickening of unknown etiology. In addition, Lee et al demonstrated that CNB has high diagnostic accuracy in differentiating malignant tumors, tuberculosis, and nonspecific inflammation, with a sensitivity of 83.3–93.3% and a specificity of 83–100% [16]. Beyond diagnosis, CNB provides crucial information for treatment planning and prognostic evaluation. Its high specificity is particularly valuable when evaluating suspicious peritoneal thickening, where accurate tissue characterization directly impacts clinical management [16].

In contrast, FNA was performed in 276 cases, accounting for approximately 23.7% (276/1165) of the total cases. Among these, 183 lesions were guided by EUS [9, 15, 17, 22], 28 by US [19, 20], and 65 by US combined with CT [11]. Interestingly, all studies that utilized EUS-guided peritoneal puncture employed FNA, and only 10 patients were found to have infiltrative peritoneal lesions [9, 22]. In cases of CT- or US-guided peritoneal puncture, FNA is primarily used for deep-seated lesions or masses that require passage through the intestinal wall. For example, Gottlieb et al identified a total of 26 peritoneal masses using US-guided FNA. They noted that probes could often be used to displace the intestine away from the needle path. However, when the intestine cannot be avoided, only FNA is feasible [19]. Alternatively, the Souza team adopted a combined approach using both CNB and FNA. In their study, the sensitivity for diagnosing benign and malignant peritoneal lesions was 91% for FNA and 93% for CNB [11].

Although both techniques are considered safe, CNB may carry a higher risk of bleeding compared to FNA [15, 17]. Among the 1165 cases included in our study, a total of 26 (approximately 2.2%) experienced complications following paracentesis, and only one case [23] resulted in tumor dissemination. While the overall complication rate was low, the single case of tumor dissemination deserves careful consideration. The reported 0.09% seeding risk likely underestimates true incidence, as most studies had follow-up periods shorter than 12 months [23].

Finally, according to the QUADAS-2 assessment, the included studies were categorized as having either a low or uncertain risk of bias. The low-risk studies (*n* = 10) demonstrated a sensitivity of 93% (91–95%), compared with 92% (88–95%) in uncertain-risk studies (*n* = 5). Conversely, the uncertain-risk studies exhibited 4% higher specificity (98% vs 94%). This inverse relationship may be attributed to the smaller number of cases analyzed and differences in the reference standards used across studies [9, 10, 18–20]. Additionally, none of the studies explicitly stated whether the pathologists were blinded to the image-guided puncture results before final diagnosis, which could have introduced bias into the subgroup analysis.

There are some limitations in our study. First, only 15 studies were included. Second, we failed to obtain unpublished data or data from studies with insufficient information, and language restrictions may further affect the reliability of the results. Third, in this meta-analysis, most studies used postoperative pathology as the reference standard for diagnosing benign and malignant peritoneal lesions [4, 11, 15, 17, 21, 22]; however, two studies used diagnostic laparoscopy as the reference standard [9, 20], and three studies included other reference standards [18, 19, 24]. Furthermore, some studies utilized FNA, while others employed CNB. Although both methods are validated for diagnosing peritoneal lesions, differences in the needle types and puncture success rates could influence the study outcomes. In conclusion, this meta-analysis demonstrates that image-guided puncture is a minimally invasive and safe technique with high diagnostic accuracy across various imaging modalities, puncture methods, and lesion types (mass or diffuse infiltration). Regarding imaging modalities, US is generally preferred for routine use given its multiplanar capability. However, for subtle diffusely infiltrative peritoneal thickening or deep-seated lesions with insufficient US visualization, combined CT/US is recommended to enhance accuracy. In patients with small nodules or suspected subtle ascites, EUS-guided FNA is a viable option. Concerning puncture methods, CNB is often preferred owing to its higher sensitivity, diagnostic accuracy, and capability in assessing diffuse peritoneal thickening. By contrast, FNA (frequently combined with EUS) is recommended for microscopic peritoneal nodules, occult ascites, or anatomically challenging lesions, though its diagnostic utility may be limited by smaller tissue samples [9]. Nevertheless, large-scale prospective multicenter studies are needed to validate the diagnostic performance of image-guided puncture in differentiating malignant from benign peritoneal lesions. Future research should focus on enhancing the diagnostic yield and spatial resolution of image-guided puncture for suspected peritoneal pathology.

## Supplementary information

Supplementary information

## Abbreviations

AUC: Area under the curve
CI: Confidence intervals
CNB: Core needle biopsy
CT: Computed tomography
DOR: Diagnostic odds ratio
EUS: Endoscopic ultrasound
FN: False negative
FNA: Fine needle aspiration
FP: False positive
QUADAS-2: Quality Assessment of Diagnostic Accuracy Studies-2
SROC: Summary receiver operating characteristic
TN: True negative
TP: True positive
US: Ultrasound
