# Supplementary material for: Image-guided puncture for differentiating malignant from benign peritoneal lesions: a systematic review and meta-analysis
Source: Eur Radiol. 2025 Nov 20;36(5):3903–15. doi: 10.1007/s00330-025-12026-w (PMC13086779; doi:10.1007/s00330-025-12026-w)
Supplement: Supplementary file 1 — Supplementary information [file 330_2025_12026_MOESM1_ESM.pdf]

Image-guided puncture for differentiating malignant from benign peritoneal lesions: a systematic review and meta-analysis

ELECTRONIC SUPPLEMENTARY MATERIAL

Supplementary Material 1 The basic content and structure of the QUADAS-2 tool

Domain	Patient Selection	Index Test	Reference Standard	Flow and Timing
**Risk of Bias Questions**	- Was a consecutive or random sample of patients enrolled?<br>- Was a case-control design avoided?<br>- Were inappropriate exclusions avoided?	- Were the index test results interpreted without knowledge of the reference standard results?<br>- If a threshold was used, was it pre-specified?	- Is the reference standard likely to correctly classify the target condition?<br>- Were the reference standard results interpreted without knowledge of the results of the index test?	- Was there an appropriate interval between the index test and reference standard?<br>- Did all patients receive the same reference standard?<br>- Were all patients included in the analysis?
**Concerns Regarding Applicability Questions**	-Are there concerns that the target condition as defined by the reference standard does not match the question?	- Are there concerns that the index test, its conduct, or interpretation differ from the review question?	- Does the reference standard, its conduct, or interpretation differ from clinical practice?	Not applicable (this domain focuses only on risk of bias).<br>|
